# Effect of copper sulfate on the external microbiota of adult common snook (*Centropomus undecimalis*)

**DOI:** 10.1186/s42523-021-00085-5

**Published:** 2021-03-02

**Authors:** Andrea M. Tarnecki, Noah J. Levi, Matthew Resley, Kevan Main

**Affiliations:** 1 Marine Immunology Program, Mote Marine Laboratory, 1600 Ken Thompson Parkway, Sarasota, FL 34236 USA; 2 Biology Department, Wabash College, 301 West Wabash Avenue, Crawfordsville, IN 47933 USA; 3 Current affiliation: Medical Scientist Training Program, University of Miami Miller School of Medicine, 1600 NW 10th Avenue, Miami, FL 33101 USA; 4 Directorate of Fisheries and Aquaculture, Mote Aquaculture Research Park, 874 WR Mote Way, Sarasota, FL 34240 USA

**Keywords:** Microbiota, Common snook, Aquaculture, Copper sulfate, External mucosa, Recirculating aquaculture system

## Abstract

**Background:**

The environment exerts a strong influence on the fish external microbiota, with lower diversity and increased abundances of opportunistic bacterial groups characterizing cultured fish compared to their wild counterparts. Deviation from a healthy external microbiota structure has been associated with increased susceptibility to bacterial pathogens. Treatment of wild-caught broodstock with copper sulfate for the removal of external parasites is a common aquaculture practice. Despite the microbiota’s importance to fish health, the effects of copper sulfate on mucosal bacterial communities and their ability to recover following this chemical treatment have not been examined. The skin microbiota of adult common snook was characterized from wild individuals (Wild), and wild-caught fish maintained in recirculating aquaculture systems (RAS) immediately following a month-long copper sulfate treatment (Captive-1), and then two-weeks (Captive-2) and 2 years (Captive-3) after cessation of copper treatment.

**Results:**

The skin microbiota of wild fish were characterized by high diversity and taxa including *Synechocococcus, SAR11*, and a member of the *Roseobacter* clade. Bacterial diversity decreased in Captive individuals during the 2-year sampling period. Captive fish harbored greater abundances of *Firmicutes*, which may reflect glycan differences between aquaculture and natural feeds. Bacterial taxa with copper resistance mechanisms and indicative of metal contamination were enriched in Captive-1 and Captive-2 fish. *Vibrionaceae* were dominant in Captive fish, particularly immediately and 2 weeks following copper treatment. Based on our observations and previous literature, our results suggest putatively beneficial taxa amass over time in captivity. Within 2 years, Captive individuals harbored *Bacillus* which contains numerous probiotic candidates and the complex carbon degraders of the family *Saprospiraceae*. Predicted butanoate metabolism exceeded that of Wild fish, and its reported roles in immunity and energy provision suggest a prebiotic effect for fishes.

**Conclusions:**

The mucosal microbiota contains bacterial taxa that may act as bioindicators of environmental pollution. Increases in mutualistic groups indicate a return to a beneficial skin microbiota following copper sulfate treatment. Our data also suggests that vastly different taxa, influenced by environmental conditions, can be associated with adult fish without noticeable health impairment, perhaps due to establishment of various mutualists to maintain fish mucosal health.

**Supplementary Information:**

The online version contains supplementary material available at 10.1186/s42523-021-00085-5.

## Background

Fish skin mucosa is the first line of defense against bacterial infections. This protective layer prevents attachment and proliferation of pathogens and provides a surface for aggregation of secreted innate immune enzymes, antimicrobial peptides, and immunoglobulins [1]. Acting as an extension of these defenses, the external mucosa maintains a diverse bacterial assemblage inhabited primarily by commensal microorganisms that help to train the fish immune system and competitively exclude pathogens [2]. Genetic factors, such as mucus composition and immune function, as well as local environmental parameters strongly influence the taxonomic structure of these bacterial communities [2–4]. Disruption of normal immune responses and microbiota structure enhance susceptibility to opportunistic bacterial pathogens normally suppressed by these mucosal defenses [5].

As the surrounding environment shapes mucosal microbiota, wild and farmed fish of the same species have distinctive bacterial community compositions [4, 6–8], with decreased bacterial diversity and increased abundance of opportunistic pathogens characterizing captive individuals. The intensive culture conditions and high organic input of aquaculture systems generate an ideal environment for proliferation of opportunistic r-strategists including potentially pathogenic bacteria. In recirculating aquaculture systems (RAS), disinfection methods such as ultraviolet light and ozone reduce competition with slower-growing, commensal K-strategists [9–11], and may intensify expansion of potentially harmful microbes. Although adult fish are less susceptible to opportunistic disease than larvae [11], aquaculture practices that decrease bacterial diversity in the fish microbiota relinquish previously inhabited niches within the mucosa that opportunists can colonize, thereby increasing contact rates and potentially harmful interactions between the fish host and aquatic pathogens.

The common snook (*Centropomus undecimalis*) is a popular sport and food fish species throughout its geographic range from Florida to Brazil. The species is sensitive to cold events, as well as harmful algal blooms, and severe occurrences lead to mortalities and the closure of this economically important fishery [12]. Current conservation efforts by the Florida Fish and Wildlife Conservation Commission (FWC) and Mote Marine Laboratory (MML) include captive rearing of snook for release to enhance stocks, requiring collection of wild adult common snook, and transitioning them to captivity for spawning. This practice includes periods of chemical treatment (i.e., copper sulfate) aimed at removing external parasites. Copper sulfate is an effective compound, commonly used in aquaculture to prevent parasitic infections and excess algal growth [13], but its impact on bacteria is not often considered. Tom-Petersen et al. [14] measured a negative impact of copper on bacterial growth; however, Qian et al. [15] reported an increase in the opportunistic genus *Vibrio* in shrimp treated with copper sulfate. Previous studies indicate that chemical treatment alters the fish skin microbiota and increases susceptibility to bacterial disease [16]. Thus, there is potential for these treatments to exacerbate alterations of the skin microbiota already induced by captivity. Due to the intimate relationship between the microbiota and fish health, understanding the influence of these treatments on the fish microbiota is crucial to maintenance of fishes in RAS.

Despite the common snook’s importance to fisheries, very little is known about the species’ microbiota. Our group recently characterized the skin-associated microbiota of juvenile common snook throughout transition from captivity to the wild during stock enhancement efforts [4]. Despite vast differences in microbial assemblages between wild and captively-reared individuals, the external microbiota adapted quickly during acclimation, and within 2 days captively-reared individuals placed in the natural environment harbored a microbiota reflective of wild-caught individuals. We have also characterized the microbiota of larval common snook during the first month of development in RAS [17, 18]. However, to our knowledge, no studies have described microbiota composition in adult common snook. The purpose of this study was to quantify, characterize, and compare external mucosal microbiota of wild and captive adult common snook following treatment with copper sulfate. We compared the external microbiota of wild snook (Wild) to broodstock transitioned into captivity at 3 sampling periods: 1) immediately following a one-month copper sulfate treatment (Captive-1), 2) 2 weeks following copper sulfate treatment (Captive-2), and 3) 2 years after copper sulfate treatment (Captive-3). These sampling points were chosen to coincide with planned spawning events at our facility, allowing for opportunistic sampling while fish were already being handled.

## Methods

Wild adult common snook were captured to build captive broodstock populations. These fish were caught at various times and locations within the coastal region of western central Florida (Table 1). Following capture, fish were transported to Mote Aquaculture Research Park (MAP), Sarasota, Florida and transitioned to captivity in RAS containing 28,000 L tanks and equipped with a 0.085 m^3^ drop filter (Aquaculture Systems Technologies, L.L.C, New Orleans, LA) for solids removal, a 900 L moving bed reactor containing 0.283 m^3^ plastic extruded floating media (AMB™ media, EEC, Blue Bell, PA) for biofiltration, a protein skimmer, two 150 W High Output SMART HO UV® units (Emperor Aquatics, Inc.®, Pottstown, PA), and 126,000 btu Aquacal chiller (AquaCal AutoPilot, Inc., St Petersburg, FL). System environmental parameters were as follows: temperature 30 ± 1 °C, salinity 35 ± 1 ppt, dissolved oxygen 5–9 mg/L, and pH 7.5–8.4. Captive fish were maintained under a photoperiod regime of 15 h Light: 9 h Dark. Upon arrival of new broodstock in June 2016, the snook were feed trained, and once all fish were eating the systems, the systems were treated with copper sulfate pentahydrate. Copper sulfate was mixed with clean seawater from the RAS following filtration to generate a stock whose concentration varied depending on copper measurements from treated broodstock tanks. Copper stock solutions were added to increase tank concentrations by 0.1 ppm per day until concentrations reached 0.3 ppm. Copper sulfate was added as needed to maintain at 0.3 ppm for 30 days. Following the month-long treatment, copper levels depurated naturally from the system.

**Table 1:** Capture dates and locations of captive broodstock sampled in this study

Fish Tag ID	Sex	Date Captured	Location Captured	Method of Capture
1272	Male	03 Jun 2016	Snake Island, Venice, FL	Hook & line
1307	Male	03 Jun 2016	Snake Island, Venice, FL	Hook & line
1270	Male	03 Jun 2016	Snake Island, Venice, FL	Hook & line
1265	Male	03 Jun 2016	Snake Island, Venice, FL	Hook & line
5D0D	Male	13 May 2015	Snake Island, Venice, FL	Hook & line
1302	Female	03 Jun 2016	Snake Island, Venice, FL	Hook & line
3C79	Female	19 Nov 2012	Bowlees Creek, Sarasota, FL	Seine net
1260	Female	03 Jun 2016	Snake Island, Venice, FL	Hook & line
7D36	Female	13 May 2015	Snake Island, Venice, FL	Hook & line
2B2B	Female	19 Nov 2012	Bowlees Creek, Sarasota, FL	Seine net

Mucus samples were collected from 10 individuals (5 males, 5 females to control for potential unknown differences between sexes) immediately following copper sulfate treatment (Captive-1; July 2016) as described below. Additional samples were collected from the same individuals 2 weeks following conclusion of copper sulfate treatment (Captive-2; August 2016). An additional sample was taken from these fish 2 years later (Captive-3; December 2018), although only 8 individuals remained due to mortality of one male and one female (5D0D and 2B2B, respectively, see Table 1) during this two-year time frame. It should be noted that fish caught prior to 2016 received previous copper sulfate treatments in our facility. The treatments prior to 2016 were at 0.2 ppm, and treatment protocols in 2016 and beyond used a target of 0.3 ppm as this concentration increased efficacy without deleterious effects on broodstock health and reproductive performance. Fish captured in 2012 received a prophylactic treatment in November 2012 and another in February 2015 to control an outbreak of the glass anemone *Aiptasia pulchella*. The 2012 fish as well as the individuals caught in 2015 received a prophylactic copper sulfate treatment in November 2015. All fish in the study received the treatment described above in July 2016. In addition to samples from Captive individuals, mucus was collected from 5 male and 5 female wild common snook, caught using seine nets at Rattlesnake Key in Terra Ceia Bay, Palmetto, Florida (27.548259 N, 82.630184 W) during the summer of 2016.

Fish were anesthetized and mucus collection for microbiota analysis took place opportunistically during reproductive sampling events, as previously described by Rhody et al. [19]. During this process, sterile plastic spatulas were used to delicately scrape mucus from the sides of each individual. As one person held the fish vertically, a second person gently scraped both sides of the fish from below the gills to the end of the anal fin, collecting the mucus in a sterile conical tube as it dripped from the fin. Volume of mucus collected ranged from 250 to 1500 μL. Samples were held on ice for further processing (~ 4 h).

Mucus was vortexed thoroughly and a sterile swab was coated with the mixed mucus for microbiota characterization using high-throughput sequencing. Swabs were stored at − 80 °C until further analysis. The use of the swab was consistent with methods previously employed in our lab to analyze common snook skin microbiota [4]. This decision was to allow comparisons with our previous study on juvenile snook [20] with as little sampling method bias as possible, as it has not been determined if freezing raw mucus alters community structure differently than freezing mucosal swabs. Bacterial counts were determined for Wild, Captive-1, and Captive-2 fishes only. Mucus remaining following swab collection was serially diluted and plated on tryptic soy agar supplemented with 2% NaCl (TSA + S) for total bacterial counts and on thiosulfate citrate bile salts sucrose agar (TCBS) for total *Vibrio* counts. Sucrose-fermenting (yellow) and non-sucrose fermenting (green) colonies were counted separately on TCBS.

DNA was extracted from mucosal swabs using the PowerSoil DNA Isolation Kit (Mo Bio Laboratories, Carlsbad, CA, USA) according to manufacturer protocols. The primers 515F/806R were used to amplify the V4 variable region of the 16S rRNA gene with the HotStarTaq Plus Master Mix Kit (Qiagen, Valencia, CA, USA). PCR conditions were as follows: 94 °C for 3 min, followed by 28 cycles of 94 °C for 30 s, 53 °C for 40 s, and 72 °C for 1 min, with a final extension at 72 °C for 5 min. Negative controls were included in PCR reactions to assess potential reagent contamination. Resulting PCR products were pooled in equal proportion based on molecular weight and DNA concentration. Pooled products were purified with calibrated Ampure XP beads. Amplicon sequencing was performed on the Illumina MiSeq platform (Illumina, Inc., San Diego, CA, USA) following standard protocols. Sequencing was performed at MR DNA (www.mrdnalab.com, Shallowater, TX, USA). Sequences were processed using the Mothur MiSeq SOP [21] accessed on 3 May 2019 in Mothur 1.42.0. Operational taxonomic units (OTUs) were identified at 97% similarity and classified using the Silva [22] and the GreenGenes [23] databases for functional predictions. OTUs were used as opposed to amplicon sequence variants (ASVs) to minimize methods bias during results comparisons with previous snook studies in our laboratory. The total number of sequences was standardized to the sample with the lowest coverage (23,592 sequences) prior to calculation of alpha diversity including species richness (i.e., number of OTUs) and evenness (Shannon Evenness Index).

Bacterial counts, richness, and evenness were analyzed using two-way ANOVA with sampling period and sex as main effects followed by Tukey’s HSD post hoc tests where significant. Data was transformed using Box-Cox transformations to meet the assumptions of normality and homogeneity of variance. Differences between microbiota were determined using permutational analysis of variance (PERMANOVA) and similarity percentages (SIMPER) in Primer v6 [24]. Linear discriminant analysis Effect Size (LEfSe) was used to identify OTUs responsible for differences between sampling periods [25] and phylogenetic investigation of communities by reconstruction of unobserved states (PICRUSt) was used to predict microbiota function [26], with both analyses occurring in Mothur v1.42.0. Significant differences in predicted Kyoto Encyclopedia of Genes and Genomes (KEGG) pathways classified using PICRUSt were determined in STAMP v2.1.3 [27] via ANOVA followed by Tukey-Kramer post-hoc tests, using a *p*-value filter of > 0.05 and an Effect size < 0.75.

As this study did not include negative kit controls to account for kit reagent contamination, OTUs identified in previous research as potential contaminants [28, 29] were removed and data analysis was repeated as described above. Excluding potential contaminant OTUs did not change overall findings, but it did alter some genus-level observations, and these are discussed herein.

## Results

### Culturable bacterial counts

Total bacterial counts were highly variable among adult common snook (Table 2). Total bacterial counts were generally higher immediately following copper sulfate treatment, but these differences were not significant [*F*_2,24_ = 0.695, *p* = 0.509]. There was no detectable influence of sex on total bacterial counts [*F*_1,24_ = 0.907, *p* = 0.351], and no interaction between sampling period and sex [*F*_2,24_ = 1.62, *p* = 0.219]. All plated dilutions on TCBS from Captive-1 individuals were too numerous to count; therefore, counts were conservatively estimated using 300 colonies per plate as follows:

$$ \frac{CFU}{mL}=\frac{\frac{CFU}{plate}\ x\  dilution\ factor}{vol. sample\ plated\ (mL)}:=\frac{300\  CFU\ x\ 5}{0.1\  mL}:=1.5\ x\ {10}^4\ \frac{CFU}{mL} $$

**Table 2:** Bacterial counts measured from adult common snook external mucus. *P* values resulting from two-way ANOVA are indicated at the bottom of the table

Sampling period	Sex	Total bacterial counts (CFU/mL)	Total Vibrio counts (CFU/mL)
Wild	Male	4.25 × 10^5^ ± 1.76 × 10^5^	1.69 × 10^3^ ± 1.00 × 10^3^
	Female	5.30 × 10^5^ ± 2.85 × 10^5^	3.89 × 10^3^ ± 1.53 × 10^3^
Captive-1	Male	2.71 × 10^6^ ± 3.83 × 10^6^	1.50 × 10^4a^
	Female	1.40 × 10^6^ ± 2.11 × 10^6^	1.50 × 10^4a^
Captive-2	Male	3.73 × 10^5^ ± 1.57 × 10^5^	3.12 × 10^5^ ± 2.65 × 10^5^
	Female	1.45 × 10^6^ ± 7.23 × 10^5^	8.46 × 10^5^ ± 5.24 × 10^5^
ANOVA	Sampling Period	*p* = 0.509	*p* < 0.001
	Sex	*p* = 0.351	*p* = 0.008
	Sampling period x Sex	*p* = 0.219	*p* = 0.866

^a^ Captive-1 Vibrio counts are estimated assuming 300 colonies per plate as the results were too numerous to count

Neither total nor *Vibrio* counts were determined for Captive-3 fish. As *Vibrio* counts in Captive-1 fish were estimated, these counts were not compared statistically with Wild or Captive-2 individuals; however, the colony counts were over 5 times higher in Captive-1 than in Wild fishes. Captive-2 fishes harbored over 200 times greater *Vibrio* counts than Wild fishes and this difference was statistically significant [*F*_1,16_ = 248, *p* < 0.001]. The data suggests a difference in *Vibrio* counts between sexes [*F*_1,16_ = 9.27, *p* = 0.008], and sampled females harbored approximately 2.7X more *Vibrio* than males. The proportion of sucrose-fermenting to non-sucrose fermenting *Vibrio* was 2.5 and 0.5 in Wild and Captive-2 fish, respectively.

### Microbiota characterization

Good’s coverage values were above 0.949 in all samples, indicating a majority (95%) of predicted OTUs in these samples were detected using high throughput sequencing. Species richness (number of OTUs) was significantly higher in Wild fish than any group of Captive fish [*F*_3,30_ = 28.5, *p* < 0.001] (Table 3). Species richness decreased over time in captivity, as significantly more OTUs were identified in Captive-1 fish than Captive-3 fish. However when potential contaminants were removed, only the differences between Wild and Captive fish remained. There was no difference detected between sexes [*F*_1,30_ = 0.103, *p* = 0.751] or for the interaction between sampling period and sex [*F*_3,30_ = 0.070, *p* = 0.975]. Wild fish had significantly greater bacterial species evenness than captive fish [*F*_3,30_ = 13.0, *p* < 0.001], with no detectable difference between sexes [*F*_1,30_ = 0.046, *p* = 0.831] or interactions between these factors [*F*_3,30_ = 0.921, *p* = 0.443].

**Table 3:** Diversity statistics within the external microbiota of adult common snook

Sampling Period	Sex	# OTUs	Shannon Evenness Index
Wild	Female	1967 ± 971	0.825 ± 0.030
Wild	Male	1840 ± 334	0.822 ± 0.026
Captive-1	Female	568 ± 136	0.611 ± 0.150
Captive-1	Male	508 ± 129	0.679 ± 0.067
Captive-2	Female	400 ± 36	0.656 ± 0.045
Captive-2	Male	447 ± 64	0.650 ± 0.025
Captive-3	Female	405 ± 29	0.671 ± 0.067
Captive-3	Male	368 ± 20	0.623 ± 0.009
ANOVA	Sampling Period	*p* < 0.001	*p* < 0.001
	Sex	*p* = 0.559	*p* = 0.426
	Sampling Period x Sex	*p* = 0.214	*p* = 0.504

**Fig. 1:**
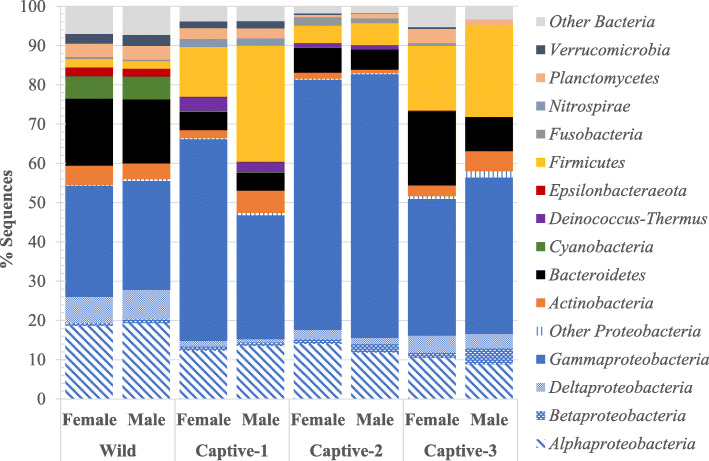
Relative abundances of phyla identified in the adult common snook external microbiota

Phylum-level classifications indicated that *Proteobacteria* dominated all snook sampled; however, sampling period impacted the relative abundances of identified phyla (Fig. 1). Wild snook harbored greater abundances of *Alphaproteobacteria*, *Deltaproteobacteria*, *Bacteroidetes*, and *Cyanobacteria* than their captive counterparts. Immediately following copper sulfate treatment (Captive-1), *Bacteroidetes* were less abundant compared to Wild fish, whereas *Gammaproteobacteria*, *Firmicutes*, and *Deinococcus-Thermus* were enriched. Two weeks later (Captive-2), *Gammaproteobacteria* made up approximately 65% of the entire mucosal microbiota. At the final sampling point 2 years later (Captive-3), *Bacteroidetes* sequences increased to levels more comparable to Wild fishes; however, the *Firmicutes* remained in high relative abundance.

**Fig. 2:**
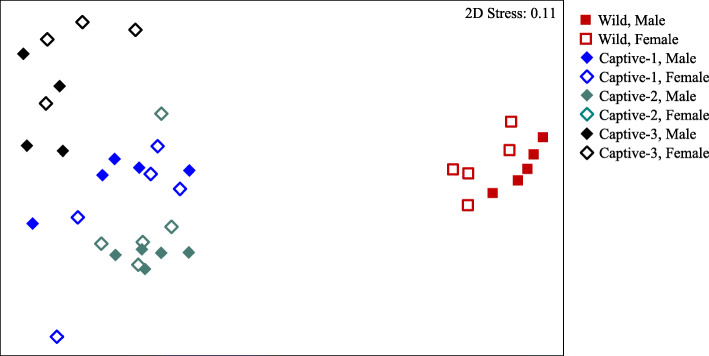
Multidimensional scaling (MDS) plot relating microbiota composition to sampling period and sex

**Fig. 3:**
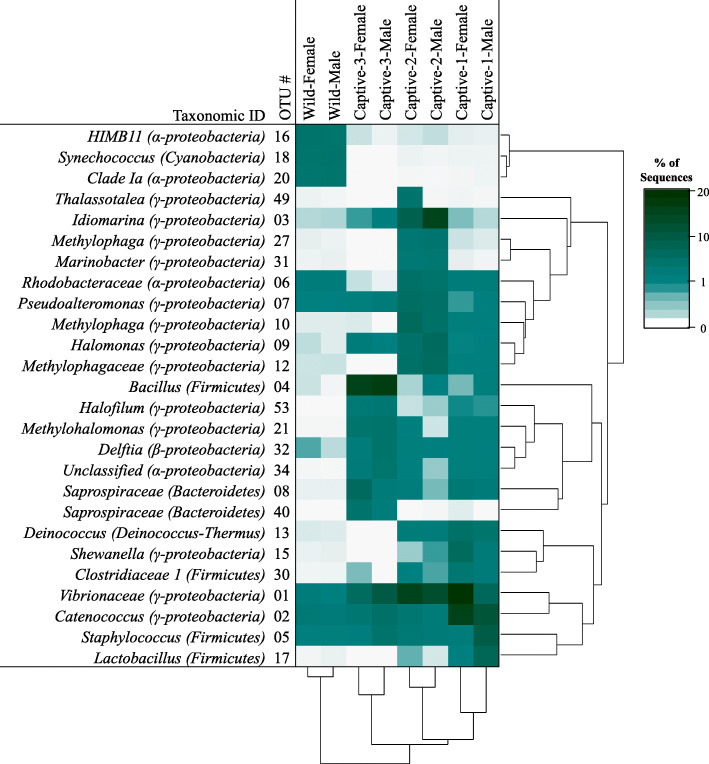
Heat map indicating relative abundance of discriminatory OTUs among sampling periods

PERMANOVA detected significant differences in external microbiota structure based on the interactions of sampling period and sex [*Pseudo-F*_3,30_ = 1.23, *p* = 0.020]. Snook from each sampling period (Wild, Captive-1, Captive-2, Captive-3) harbored unique microbiota [*p* (perm) ≤ 0.013] (Fig. 2). LEfSe identified 3 OTUs enriched in Wild, 6 OTUs enriched in Captive-1, 10 OTUs enriched in Captive-2, and 7 OTUs enriched in Captive-3 (LDA > 4; see Additional file 1). Some of the most abundant (> 5% total sequences) of these indicative OTUs included *Catenococcus* in Captive-1, *Vibrionaceae* and *Idiomarina* in Captive-2, and *Bacillus* in Captive-3 (Fig. 3). Apparent differences between sexes were only seen in Captive-1 fish [*p* (perm) = 0.030]. OTUs enriched in males were primarily within the *Firmicutes* and included the genera *Staphylococcus*, *Lactobacillus*, and *Chryseomicrobium*, whereas OTUs increased in females included *Gammaproteobacteria* (*Vibrionaceae*, *Catenococcus*, and *Shewanella*) and *Deinococcus-Thermus* (*Deinococcus*).

When potential contaminating OTUs were removed from analysis, there was no detectable influence of sex or its interaction with sampling period on total microbiota structure, but the main effect of sampling period remained (*Pseudo-F*_3,30_ = 11.417, *p* = 0.001). Although the general pattern of differences between all sampling periods remained, some changes occurred in the LEfSe results (see Additional File 4). The taxa *Deinococcus*, *Staphylococcus*, and *Lactobacillus* were removed from Captive-1 and *Delftia* and *Bacillus* were removed from Captive-3. Discriminatory taxa identified upon reanalysis included: *AEGEAN-169 marine group* enriched in Wild; *Rhodobacteraceae* and *Cetobacterium* enriched in Captive-2; and *Aquisalimonas*, *Fodinibius*, and an unclassified Alphaproteobacterium in Captive-3.

**Fig. 4:**
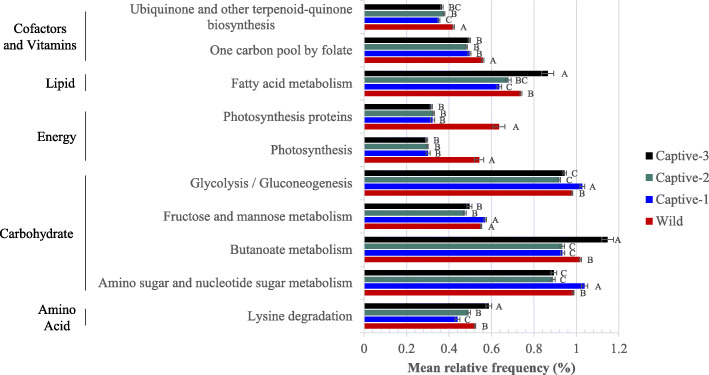
Predicted microbiota function by sampling period. Only pathways with a frequency of at least 0.5% are included

Less than 5% of total bacterial sequences from Wild fish fell within taxa that contain potential fish pathogens as listed in Austin and Austin [30] (see Additional file 2), with a majority of these classified as *Vibrionaceae*, *Arcobacter*, and *Acinetobacter*. Potentially pathogenic taxa composed 25–30% of Captive microbiota with *Vibrionaceae* highly abundant at all three sampling points. *Clostridium* and *Lactobacillus* (both potential contaminants) were primarily identified in Captive-1, *Halomonas* and *Pseudoalteromonas* primarily in Captive-2, and *Bacillus* and *Micrococcus* (both potential contaminants) primarily in Captive-3. Although some of the taxa are identified as potential contaminants, they have been reported in culture-based studies from the skin/gill microbiota of fishes [31–34] and therefore may be true representatives in the community.

*Predicted Microbiota Function.* PICRUSt analysis identified 27 pathways that were significantly altered among sampling periods, with 12 pathways having mean relative frequencies of at least 0.5% (Fig. 4), all of which fell within Level 1 Metabolism. Pathways higher in Wild fish included metabolism of terpenoids and polyketides (terpenoid backbone biosynthesis), cofactors and vitamins (one carbon pool by folate) and energy metabolism (photosynthesis, photosynthesis-antenna proteins, and photosynthesis proteins). Lipid metabolism (fatty acid metabolism) and amino acid metabolism (lysine degradation) were enriched in Captive-3 individuals. Butanoate metabolism, propanoate metabolism, and carbon fixation pathways in prokaryotes were highest in Captive-3 fish followed by Wild fishes, whereas amino sugar and nucleotide sugar metabolism and glycolysis/gluconeogenesis was increased in Captive-1 fishes followed by Wild fishes. Fructose and mannose metabolism was more abundant in Wild and Captive-1 fishes than subsequent sampling periods.

## Discussion

The use of copper sulfate to remove external parasites is a common aquaculture practice. This study demonstrated that copper sulfate treatment, in combination with the RAS environment, alters the external microbiota of adult common snook as compared to their wild counterparts. We could not separate microbiota impacts from copper sulfate versus captivity alone as all fish in captivity were treated with the chemical, but changes in communities during depuration of copper from the system and enrichment of particular taxa (described below) indicate a chemical treatment effect.

We did not detect a difference in total bacterial concentrations between Wild and Captive fishes using viable plate counts (TSA + S); however, TCBS counts indicated Captive-1 and Captive-2 mucus contained at least 5X and 200X more *Vibrio*, respectively, than Wild individuals. This increase was confirmed using high-throughput sequencing as Captive-1 and Captive-2 fish averaged 11.5% of sequences identified as *Vibrionaceae* versus 1% in Wild fish. *Vibrio* were more abundant in captive juvenile common snook than wild and wild-acclimated fish [4]; thus, RAS select for *Vibrio* in the fish skin microbiota. Increased nutrient input may allow for greater proliferation of these r-strategists, while ultraviolet light, ozone, and copper sulfate reduced bacterial competition for space and nutrients. Some fish exposed to copper exhibit immunosuppression and increased susceptibility to *Vibrio* pathogens [35, 36]. High (40 ppm) concentrations of copper reduce virulence in some vibrios [37], whereas lower concentrations trigger production of copper detoxifying compounds, enabling copper resistance [38] that allows colonization, immune avoidance, and lysis of host immune cells, thereby enhancing virulence of the microbe [39]. Copper resistance mechanisms in the *Vibrionaceae* may explain their enrichment and persistence in copper sulfate-treated fish. As copper may induce virulence, it is vital to maintain a copper level that is non-toxic to the fish to prevent immunosuppression and other physiological damage [13] that can increase fish disease susceptibility. Culturing on TCBS indicated a decrease in the relative abundance of sucrose-fermenting *Vibrio* in Captive fish as compared to Wild fish. As sucrose is a disaccharide composed of glucose and fructose, a reduction in the proportion of bacteria capable of fermenting this sugar supports the PICRUSt results indicating a decrease in mean relative frequency of glycolysis and fructose/mannose metabolism in Captive fish. It should be noted that the salt concentration in the media used in this study was not equal to the salinity of the rearing environment of the fish and this likely influenced the number of cultivable bacteria and *Vibrio*, as well as the proportion of sucrose fermenters. Oliver et al. [40] did not find a correlation between total bacterial counts, total *Vibrio* counts, or percent sucrose fermenters and salinity in water. However, there was a negative correlation between total bacteria and salinity in plankton samples, as well as a negative correlation between sucrose fermenters and salinity in oysters. Other studies indicate a positive correlation between total *Vibrio* counts and salinity [41, 42]. As our samples were host-associated, we may have counted fewer bacteria and sucrose fermenters if the salinity of the media used was 3.5%.

Total *Vibrio* plate counts and sequencing data suggest females may harbor more vibrios than males in Captive-1 and Captive-2 individuals, but the cause is unclear. The scraping technique used to collect mucus may amass greater amounts of water from larger individuals (Captive females were 3.5 ± 2.7 kg, 66.4 ± 19.3 cm and Captive males were 0.8 ± 0.2 kg, 45.4 ± 3.3 cm) and future studies should standardize counts to protein concentration to account for this variation. Literature does suggest a potential role of immune response in the differing vibrio abundances between sexes, as male sea bass (*Dicentrarchus labrax*) launched a greater IgM response to injected heat-inactivated *Vibrio anguillarum* than females [43]. If this stronger antibody defense occurs in common snook, males may be better equipped to prevent *Vibrio* colonization within the mucosal microbiota. A formal study designed to detect differences between sexes would be required to determine if this is a true pattern or a random sampling effect resulting from small sample size.

RAS decreased bacterial diversity and evenness in the snook external microbiota compared to Wild environments, a trend that is commonly reported [4, 44–47]. It is clear that conditions in captivity influence host-associated microbial communities, in part from increased nutrient input and water disinfection as described previously, but also from altered diets which can influence both the gut and skin microbiota [48]. The phylum *Bacteroidetes* was greatly reduced following copper sulfate treatment then increased over time in captivity, nearly recovering to levels measured in Wild fish. The *Bacteroidetes* were replaced primarily with *Firmicutes* which remained abundant throughout the sampling periods. *Firmicutes* and *Bacteroidetes* play important roles in polysaccharide catabolism; however, *Bacteroidetes* are generalists for many simple and complex glycans as compared to the more specialized *Firmicutes* [49]. The gastrointestinal tract of cultured yellowtail kingfish (*Seriola lalandi*) and Atlantic salmon (*Salmo salar*) contained greater abundances of *Firmicutes* than their wild counterparts [7, 50] and it was suggested that selection for *Firmicutes* during captivity represents the limited diet encountered in aquaculture systems [50]. As diet alters the gut and skin microbiota [48], the availability of a smaller variety of polysaccharides in captive diets may be reflected in the fish skin microbiota. Copper treatment could also select for *Firmicutes* as Gram-positive bacteria have high resistance to copper toxicity [51]. Thus, our data indicates the potential for diet and copper sulfate together to influence the skin microbiota at a high taxonomic level.

As demonstrated in previous studies, the bacteria inhabiting the surrounding water likely influenced the skin microbiota composition of adult snook [47, 52], but the variability attributable to changes in water communities cannot be determined as concurrent water samples were only taken at the Captive-3 sampling period (see Additional info 3). Previous studies have indicated that aquaculture systems alter water microbial community composition [9, 47, 50], and it is likely that water microbiota change over time in RAS [18, 53] even without chemical treatment. Therefore, we recognize that there was likely a random water effect that influenced the fish skin microbiota in this study. However, samples from Captive-3 and other studies [47, 52, 54, 55] indicate the mucosal microbiota harbors a unique bacterial composition in comparison to the surrounding environment in RAS and natural environments that is influenced by host parameters including mucosal composition and host immune response as well as contaminants. Therefore, we suggest the observed changes among environments and sampling periods is not explained solely by random water effects, but also by copper sulfate and unmeasured physiological changes that occur to fish in captivity.

The greater abundance of *Cyanobacteria* in Wild fish is likely explained by the high abundance of *Synechococcus* in marine environments [56] and its ability to adapt to lower salinities found in bays [57]. Our snook, sampled from Terra Ceia Bay, FL, are likely to encounter and associate with this genus. Also abundant in Wild fish was *HIMB11* of the *Roseobacter* clade (*Alphaproteobacteria*) which is enriched in waters during blooms of *Synechococcus* [58], perhaps explaining the co-occurrence of these two groups. Although *Synechococcus* and *Clade Ia* (*SAR11*, *Alphaproteobacteria*) appear as transient groups that decreased over time in our RAS, *HIMB11* remained at low abundances in Captive fish and may represent a core member of the snook microbiota, using a wide variety of compounds for energy [59] to adjust to differing environmental conditions.

Copper sulfate treatment selected for metal-tolerant bacteria, and bacterial biomarkers of the fish mucosal microbiota may act as indicators of heavy metal pollution as suggested by Montenegro et al. [60]. *Catenococcus* (enriched in Captive-1) and *Halomonas* and *Marinobacter* (enriched in Captive-2) were found in high abundances downstream of a metal-polluted estuary where many copper tolerant isolates were collected [61] and may be bioindicators of copper contamination. Additionally, the genera *Halomonas* and *Marinobacter* positively correlate with environmental pollutants, such as polycyclic aromatic hydrocarbons and metals [60] and perform denitrification using a nitrite reductase (nirK) that requires and is positively correlated with copper [62]. *Shewanella* were abundant in Captive-1 and decreased over time in captivity. Members of this genus express copper resistance genes [63] and bioabsorb copper ions [64], perhaps leading to its enrichment immediately post treatment. *Deinococcus*, known for tolerance to high levels of radiation and oxidation, followed a similar pattern to *Shewanella*. Genes that regulate copper homeostasis in *Deinococcus* also provide protection against oxidative stress [65], and may allow survival at early captive sampling points when copper levels were still relatively high. Due to its presence in negative controls in other studies [28], we cannot rule out the possibility that the sequences attributed to *Deinococcus* are contaminants obtained during sample processing. Upon reanalysis after removing potential contaminants, *Cetobacterium* was enriched in Captive-2. This genus increased in the gut microbiota of common carp following 8 weeks of copper exposure [66] suggesting it may be copper resistant. Methylotrophic bacteria were indicative of Captive-2 fish, including two OTUs identified as *Methylophaga* and one within the family *Methylophagaceae*. The Methylophaga bacteria use amicyanin, a copper-containing protein, as an electron acceptor during degradation of methylamines [67], and these microbes may aid in removal of copper during chemical depuration. Selection for the aforementioned taxa suggests reflection of environmental pollution within the fish mucosal microbiota, and due to the intimate relationship between the microbiota and immune defenses, these microorganisms may serve as non-lethal biomarkers for contaminant exposure and fish health.

Despite vast taxonomic differences between Wild and Captive-3 mucosal microbiota, predicted functionality better resembled wild-type following time in captivity. The increase in *Bacteroidetes* in Captive-3 fishes was defined by the family *Saprospiraceae*, capable of degrading complex carbon sources [68]. Also enriched in Captive-3 individuals were members of the genus *Bacillus*, which are often used as probiotics in fishes to increase disease resistance and boost immune function [18, 69] and likely represent commensal or mutualistic members of the community as they are often isolated from apparently healthy fishes [31–33]. Butanoate metabolism, which was decreased following copper sulfate treatment, recovered to and exceeded that of wild individuals after 2 years in captivity. Butanoate is a short chain fatty acid well-recognized for its prebiotic effects in cultured fish as it provides energy to cells, boosts immune response [70, 71], and increases amino acid availability [72], relating to greater lysine metabolism detected in Captive-3 snook. It is important to point out that results from PICRUSt analysis are predictive and therefore should be interpreted as hypotheses [26, 73]. The ability to predict community function using this analysis is limited by the genomic information currently available for each taxon. In addition, it assumes that the genes are transcribed and translated. In order to test the hypotheses generated during PICRUSt analysis, the presence of the functional genes should be confirmed with metagenomics. Their expression in the community can be investigated using transcriptomics, and their translation into functional proteins investigated with proteomics. Formal studies should be conducted using these technologies to confirm the restoration of beneficial community function following copper sulfate treatment.

Although the specific members of the skin microbiota vary considerably upon transition to captivity, predictive functional redundancy within the microbial communities may allow recovery of mutualistic microbes and metabolic pathways vital to fish health. These shifts indicate that, despite the lower diversity and greatly altered taxonomic structure of captive snook external bacterial assemblages, a microbiota capable of degrading complex carbon sources, competitively excluding pathogens, boosting immunity, and producing beneficial short chain fatty acids is present in captive broodstock. The beneficial potential of the captive microbiota and the long-term survival and reproductive success of common snook broodstock suggest major alterations of the fish microbiota can occur without obvious detriments to fish health in RAS.

Currently, at least 20 fish species are grown in aquaculture for stocking purposes, with greater than 3.5 billion individuals released in 2018 [74]. In order to maintain genetic diversity and fitness upon release, it is recommended that local, wild broodstock be regularly added to the captive breeding population [75]. Therefore, maintaining fish health during transition of wild individuals to captive environments is an ongoing concern for these programs. As mucosal microbiota play a vital role in fish health via competitive exclusion of pathogens and stimulation of immune function, unraveling the connections between aquaculture practices and microbiota structure and function can help farmers anticipate and prevent potential health issues associated with captivity [76]. Microbiota alterations at the parent level could influence the bacterial communities associated with eggs and larvae through vertical transmission of bacteria [77]. As early microbial assemblages play a role in tissue development [78], immune function [79], and behavior [80], a captive microbiota may influence physiology of the fish and impact their ability to survive upon release. It is also possible that different microbiota structures result in similar physiological development, perhaps due to host genetic factors and/or functional redundancy among bacteria, and these drastic community changes do not negatively impact the individual. In order to increase understanding of host-microbe relationships in aquaculture, we recommend future studies that incorporate –omics technologies to address microbiota function and host physiology during transition from wild to captivity, as well as the impacts of a captive microbiota on larval development.

## Supplementary Information

**Additional file 1.** LEfSe results indicating OTUs discriminatory for each sampling period. Average and standard error of the mean are included for each OTU identified by LEfSe analysis as discriminatory between sampling periods. This file includes results from original analysis (‘Original Data’ tab) and analysis after removal of potential contaminants (‘Potential Contaminants Removed’ tab).

**Additional file 2.** Bacterial taxa identified in this study that contain potential fish pathogens. Average and standard error of the mean are included for each potentially pathogenic bacterial genus present in sequencing analysis, listed by sampling period. Taxa included were determined using the following source: B. Austin and D. A. Austin. 2012. Bacterial Fish Pathogens: Disease of Farmed and Wild Fish, 5th edition. Springer, New York. 652 pp.

**Additional file 3.** Data analysis files including water samples taken during sampling period Captive-3 and including year of capture for captive snook. A. Relative abundance of phyla; B. Multidimensional scaling (MDS) plot; C. Heat map indicating relative abundance of discriminatory OTUs.

**Additional file 4.** Data analysis files excluding potential contaminants. A. Relative abundance of phyla; B. Muldimensional scaling (MDS) plot; C. Heat map indicating relative abundance of discriminatory OTUs.

## Data Availability

The microbiota sequence data generated during the current study are available in the Sequence Read Archive repository (www.ncbi.nlm.nih.gov/sra/), SRA study accession PRJNA664785.

## Abbreviations

KEGG: Kyoto Encyclopedia of Genes and Genomes
LEfSe: Linear discriminant effect size
OTU: Operational taxonomic unit
PERMANOVA: Permutational analysis of variance
PICRUSt: Phylogenetic investigation of communities by reconstruction of unobserved states
RAS: Recirculating aquaculture system
SIMPER: Similarity percentages
TCBS: Thiosulfate citrate bile salts sucrose
TSA + S: Tryptic soy agar supplemented with 2% NaCl
