# Supplementary material for: Effect of copper sulfate on the external microbiota of adult common snook (Centropomus undecimalis)
Source: Anim Microbiome. 2021 Mar 2;3:21. doi: 10.1186/s42523-021-00085-5 (PMC7923503; doi:10.1186/s42523-021-00085-5)
Supplement: Supplementary file 3 — Additional file 3. Data analysis files including water samples taken during sampling period Captive-3 and including year of capture for captive snook. A. Relative abundance of phyla; B. Multidimensional scaling (MDS) plot; C. Heat map indicating relative abundance of discriminatory OTUs. [file 42523_2021_85_MOESM3_ESM.docx]

Additional file 3A. Relative abundance of phyla identified in Wild and Captive common snook skin microbiota, including water microbiota from Captive-3 sampling period.

2016

2012

2015

2D Stress: 0.12

Wild

Captive-2

Captive-3

Water

Captive-1

Additional file 3B. Multidimensional Scaling plot including water and snook skin microbiota. Sampling periods and sample type are indicated by colors whereas shapes indicate the year the snook was caught.

Additional file 3C. Heat map including water microbiota from Captive-3 sampling period and fish microbiota broken down by capture date.

| Taxonomic ID | OTU # | Wild-Female | Wild-Male | 2012:Captive-1 | 2015:Captive-1 | 2016:Captive-1 | 2012:Captive-2 | 2015:Captive-2 | 2016:Captive-2 | 2012:Captive-3 | 2015:Captive-3 | 2016:Captive-3 | Water: Captive-3 |
| --- | --- | --- | --- | --- | --- | --- | --- | --- | --- | --- | --- | --- | --- |
| HIMB11 (α-proteobacteria) | 16 |  |  |  |  |  |  |  |  |  |  |  |  |
| *Synechococcus* (Cyanobacteria) | 18 |  |  |  |  |  |  |  |  |  |  |  |  |
| Clade Ia (α-proteobacteria) | 20 |  |  |  |  |  |  |  |  |  |  |  |  |
| *Thalassotalea* (γ-proteobacteria) | 49 |  |  |  |  |  |  |  |  |  |  |  |  |
| *Idiomarina* (γ-proteobacteria) | 03 |  |  |  |  |  |  |  |  |  |  |  |  |
| *Methylophaga* (γ-proteobacteria) | 27 |  |  |  |  |  |  |  |  |  |  |  |  |
| *Marinobacter* (γ-proteobacteria) | 31 |  |  |  |  |  |  |  |  |  |  |  |  |
| Rhodobacteraceae (α-proteobacteria) | 06 |  |  |  |  |  |  |  |  |  |  |  |  |
| *Pseudoalteromonas* (γ-proteobacteria) | 07 |  |  |  |  |  |  |  |  |  |  |  |  |
| *Methylophaga* (γ-proteobacteria) | 10 |  |  |  |  |  |  |  |  |  |  |  |  |
| *Halomonas* (γ-proteobacteria) | 09 |  |  |  |  |  |  |  |  |  |  |  |  |
| Methylophagaceae (γ-proteobacteria) | 12 |  |  |  |  |  |  |  |  |  |  |  |  |
| *Bacillus* (Firmicutes) | 04 |  |  |  |  |  |  |  |  |  |  |  |  |
| *Halofilum* (γ-proteobacteria) | 53 |  |  |  |  |  |  |  |  |  |  |  |  |
| *Methylhalomonas* (γ-proteobacteria) | 21 |  |  |  |  |  |  |  |  |  |  |  |  |
| *Delftia* (β-proteobacteria) | 32 |  |  |  |  |  |  |  |  |  |  |  |  |
| Unclassified (α-proteobacteria) | 34 |  |  |  |  |  |  |  |  |  |  |  |  |
| Saprospiraceae (Bacteroidetes) | 08 |  |  |  |  |  |  |  |  |  |  |  |  |
| Saprospiraceae (Bacteroidetes) | 40 |  |  |  |  |  |  |  |  |  |  |  |  |
| *Deinococcus* (Deinococcus-Thermus) | 13 |  |  |  |  |  |  |  |  |  |  |  |  |
| *Shewanella* (γ-proteobacteria) | 15 |  |  |  |  |  |  |  |  |  |  |  |  |
| Clostridiaceae 1 (Firmicutes) | 30 |  |  |  |  |  |  |  |  |  |  |  |  |
| Vibrionaceae (γ-proteobacteria) | 01 |  |  |  |  |  |  |  |  |  |  |  |  |
| *Catenococcus* (γ-proteobacteria) | 02 |  |  |  |  |  |  |  |  |  |  |  |  |
| *Staphylococcus* (Firmicutes) | 05 |  |  |  |  |  |  |  |  |  |  |  |  |
| *Lactobacillus* (Firmicutes) | 17 |  |  |  |  |  |  |  |  |  |  |  |  |
