# Supplementary material for: Effect of copper sulfate on the external microbiota of adult common snook (Centropomus undecimalis)
Source: Anim Microbiome. 2021 Mar 2;3:21. doi: 10.1186/s42523-021-00085-5 (PMC7923503; doi:10.1186/s42523-021-00085-5)
Supplement: Supplementary file 4 — Additional file 4. Data analysis files excluding potential contaminants. A. Relative abundance of phyla; B. Muldimensional scaling (MDS) plot; C. Heat map indicating relative abundance of discriminatory OTUs. [file 42523_2021_85_MOESM4_ESM.docx]

Additional file 4A. Relative abundance of phyla identified in Wild and Captive common snook skin microbiota, excluding potential contaminant OTUs.


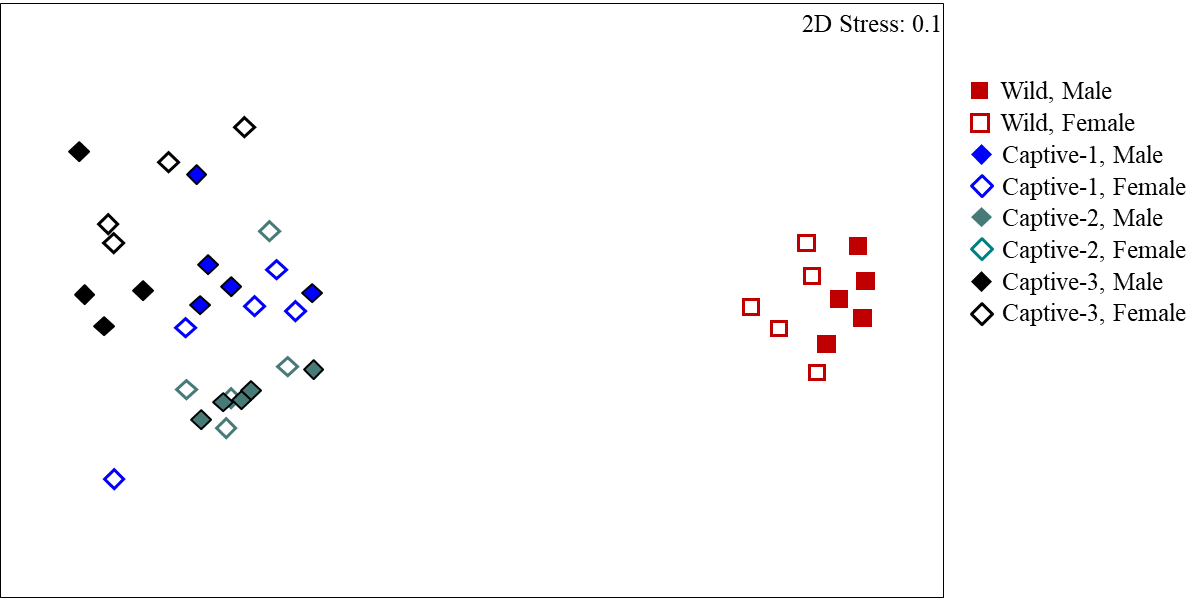


Additional file 4B. Multidimensional Scaling plot of snook skin microbiota excluding potential contaminant OTUs.

|  | OTU | Wild-Female | Wild-Male | Captive-3-Female | Captive-3-Male | Captive-1-Female | Captive-1-Male | Captive-2-Female | Captive-2-Male |
| --- | --- | --- | --- | --- | --- | --- | --- | --- | --- |
| AEGEAN-169 (α-proteobacteria) | 024 |  |  |  |  |  |  |  |  |
| HIMB11 (α-proteobacteria) | 016 |  |  |  |  |  |  |  |  |
| Clade Ia (α-proteobacteria) | 020 |  |  |  |  |  |  |  |  |
| *Synechococcus* (Cyanobacteria) | 018 |  |  |  |  |  |  |  |  |
| Saprospiraceae (Bacteroidetes) | 008 |  |  |  |  |  |  |  |  |
| *Halofilum* (γ-proteobacteria) | 053 |  |  |  |  |  |  |  |  |
| *Methylhalomonas* (γ-proteobacteria) | 021 |  |  |  |  |  |  |  |  |
| Unclassified (α-proteobacteria) | 034 |  |  |  |  |  |  |  |  |
| Unclassified (α-proteobacteria) | 101 |  |  |  |  |  |  |  |  |
| Fodinibius (Bacteroidetes) | 062 |  |  |  |  |  |  |  |  |
| Aquisalimonas (γ-proteobacteria) | 045 |  |  |  |  |  |  |  |  |
| Saprospiraceae (Bacteroidetes) | 040 |  |  |  |  |  |  |  |  |
| *Shewanella* (γ-proteobacteria) | 015 |  |  |  |  |  |  |  |  |
| Clostridiaceae 1 (Firmicutes) | 030 |  |  |  |  |  |  |  |  |
| Catenococcus (γ-proteobacteria) | 002 |  |  |  |  |  |  |  |  |
| Vibrionaceae (γ-proteobacteria) | 001 |  |  |  |  |  |  |  |  |
| *Idiomarina* (γ-proteobacteria) | 003 |  |  |  |  |  |  |  |  |
| *Pseudoalteromonas* (γ-proteobacteria) | 007 |  |  |  |  |  |  |  |  |
| *Methylophaga* (γ-proteobacteria) | 010 |  |  |  |  |  |  |  |  |
| Methylophagaceae (γ-proteobacteria) | 012 |  |  |  |  |  |  |  |  |
| *Halomonas* (γ-proteobacteria) | 009 |  |  |  |  |  |  |  |  |
| Rhodobacteraceae (α-proteobacteria) | 006 |  |  |  |  |  |  |  |  |
| Rhodobacteraceae (α-proteobacteria) | 014 |  |  |  |  |  |  |  |  |
| *Thalassotalea* (γ-proteobacteria) | 049 |  |  |  |  |  |  |  |  |
| *Cetobacterium* (Fusobacteria) | 044 |  |  |  |  |  |  |  |  |
| *Methylophaga* (γ-proteobacteria) | 027 |  |  |  |  |  |  |  |  |
| *Marinobacter* (γ-proteobacteria) | 031 |  |  |  |  |  |  |  |  |

Additional file 4C. Heat map of fish microbiota excluding potential contaminant OTUs.
